# Polishing of Monolithic Zirconia Crowns—Results of Different Dental Practitioner Groups

**DOI:** 10.3390/dj5040030

**Published:** 2017-11-14

**Authors:** Carla Kozmacs, Britta Hollmann, Wolfgang H. Arnold, Ella Naumova, Andree Piwowarczyk

**Affiliations:** 1 Department of Prosthodontics and Dental Technology, School of Dentistry, Witten/Herdecke University, Alfred-Herrhausen-Straße 44, 58455 Witten, Germany; Andree.Piwowarczyk@uni-wh.de; 2 Private Practice, 50670 Köln, Germany; BrittaHollmann@gmx.de; 3 Department of Biological and Material Sciences in Dentistry, School of Dentistry, Witten/Herdecke University, Alfred-Herrhausen-Straße 44, 58455 Witten, Germany; Wolfgang.Arnold@uni-wh.de (W.H.A.); Ella.Naumova@uni-wh.de (E.N.)

**Keywords:** dental materials, monolithic zirconia, abrasion, ceramic, polishing, surface roughness

## Abstract

This pilot study evaluates the surface roughness of monolithic zirconia crowns after chairside polishing by different dental practitioner groups. Four practitioner groups (group I: dental clinical students (*n* = 6); group II: dentists < 2 years post-qualification experience (*n* = 6); group III: dentists > 2 years post-qualification experience (*n* = 6) and group IV: dental technicians (*n* = 6)) were asked to polish two CAD/CAM-fabricated full-contour zirconia crowns (48 in total). A two-step zirconia polishing kit was used in both trials. The first trial (T1) was conducted without a time limitation. In the second trial (T2), the polish was restricted to 15 seconds for each polisher. Two blinded investigators (I1 and I2) analyzed the surface roughness (Ra) before and after polishing (Alicona measuring system). No statistically significant difference in surface roughness was found between the polishing results of the dental practitioner groups. Major difference in surface finish was achieved by dental technicians, with a median value of 25.4 nm (interquartile range 10.15–35.26 nm) for I1 in T1. The lowest difference was achieved by dental students, with a median value of Ra = 6.72 nm (interquartile range 4.7–17.9 nm) in T1. In T2, experienced dentists showed the highest difference in surface finish, with a median value of 41.35 nm (interquartile range 7.77–54.11). No significant correlation was found between polishing time and polishing results. The polishing of monolithic zirconium dioxide crowns can be performed with the present polishing set directly chairside after occlusal adjustment, regardless of the practitioner’s experience level.

## 1. Introduction

New digital technology and the growing aesthetic expectations of patients are responsible for the increasing progress in ceramic materials [1]. Metal-ceramic restorations have been the gold standard for fixed partial dentures for the past several decades [2], but they have never been able to imitate the natural translucency of dental enamel [1,3,4]. However, the range of indications for initial glass matrix ceramics was relatively small, limited to single tooth restorations [5]. The development of high-performance zirconium dioxide ceramics and the establishment of CAD/CAM (computer-aided design/computer-aided manufacturing) technology can compensate for this limitation and replace the conventional metal-ceramic restorations.

Zirconia was introduced as an advanced technical ceramic in dentistry and the development of CAD/CAM technology allows for reproducible, rapid, and relatively cheap fabrication. With its high strength value, Y-TZP (yttria tetragonal zirconia polycrystals) zirconia restorations can fill the indication gaps of the pre-existing silicate ceramics and replace metal restorations [6,7,8]. Initially, zirconia frameworks were veneered with silicate ceramic. High chipping rates of these veneered frames were observed in numerous zirconia studies, especially on posterior molars [9,10]. Consequently, to avoid chipped veneered zirconia frames, monolithic full-contour restorations were tested in less visible areas with a focus on the surface treatment [11,12,13]. Increasing antagonist wear due to the greater hardness and toughness of this ceramic material was expected [14]. Various studies disproved these expectations depending on proper surface finishing [7,9,14,15,16]. The surface condition of zirconia crowns was determined to have a substantial effect on the antagonist’s wear, indicating a need for proper surface finishing [17]. Surface finishing can be accomplished by either polishing or glazing. The literature is divided regarding surface roughness and antagonist wear after glazing and polishing. However, only studies that considered mastication demonstrated greater efficacy of surface polishing with respect to avoiding antagonist wear [14,15]. Nevertheless, the final adjustment at the dental office results in a loss of ceramic glaze, which requires re-polishing of the zirconia surface [18,19]. Sethi et al. showed that a final manual chairside polishing after occlusal adjustment exhibits no significant differences compared to a re-glazing [20]. 

Many different chairside polishing systems are available [18], but knowledge is deficient with respect to their operation, whether to use water cooling or not, or how many steps are necessary. An investigation from 2015 focused on a comparison of the effectiveness of different polishing kits [18]. The authors summarize the effectiveness of nearly every polishing kit when every sequential polishing step is applied.

In contrast to industrial polishing processes, the final chairside polishing is performed manually. Therefore, parameters such as pressure, duration, and cooling cannot easily be standardized. In order to ensure a standardized procedure that can be transferred to clinical practice, the participants used the same polishing kit with the same revolutions (6000 rpm) and duration instruction (T2). Accordingly, in the present study, whether different dental practitioners could achieve significantly different polishing results using the same polishing kit was investigated. 

We sought to determine if the variation in surface roughness is considerable and if extensive equilibration of the polishing process is warranted.

The goal of this study was to evaluate the surface roughness of monolithic zirconia crowns after chairside polishing by different dental practitioner groups relative to different duration instructions. The working hypothesis proposes significant differences in the polishing results of different practitioner groups. Second, whether or not polishing time influences the results should also be evaluated.

## 2. Materials and Methods

### 2.1. Preparation of the Specimens

Forty-eight full-contour zirconia crowns served as specimens. These specimens were CAD/CAM fabricated using 3% yttrium-stabilized Lava Plus High Translucency Zirconia Discs (3M Oral Care, Seefeld, Germany). The zirconia crowns were milled and then sintered (HT Speed MIHM-Vogt, Stutensee, Germany). After sintering, the zirconia ceramic crowns were successively ground and polished in four steps by one dentist. In the first two steps, blue and white diamond polishers were used (6000 rpm; 94018C 104 055/94018F 104 055; Komet Dental, Lemgo, Germany). In the last two steps, diamond polishing paste with a goat hair and felt brush was used (15,000 rpm; Diamond polishing paste 9300; Komet Dental, Lemgo, Germany). The occlusal adjustment was accomplished by one dentist on the mesiobuccal cusp with five strokes (parallel to cusp inclination) with a red ring diamond bur (20,000/min; REF 8379 204 023; Komet Dental, Lemgo, Germany).

### 2.2. Surface Roughness Determination

The surface roughness (roughness average, Ra) of the full-contour zirconia crowns was analyzed using the optical profilometry Infinite Focus System G3 (Alicona Imaging, Graz, Austria). Roughness measurements were taken before and after occlusal adjustment and after polishing with a vertical resolution of 450 nm and an *Lc* value of 25 μm. The Lc is the spatial frequency value for the high-pass filter applied on the profiles, which is used to cut-off the environmental noise affecting the measurements. From past experience, the *Lc* value of 25 μm has been proven to be best value for this kind of surface [21]. Ten linear measurements and 10 area measurements were performed to determine the *Ra*-(mean value of roughness) [22]. Within the enlarged 510 × 510 μm section of the machined surface, linear measurements of 100 μm in length and surface measurements of 50 × 50 μm^2^ were performed. The surface measurement followed the ISO 11562 recommendations to guarantee the reproducibility of the results. All surface measurements were performed by two blinded investigators independently. 

### 2.3. Selection/Composition of the Practitioner Groups

The 24 participants were chosen randomly from 4 different practitioner groups. Eighteen participants were dental students and dental staff of the University Witten/Herdecke:

I: clinical dental students (*n* = 6),
II: dentists > 2 years of post-qualification experience (*n* = 6),
III: dentists < 2 years of post-qualification experience (*n* = 6) and six participants were technicians from a commercial local laboratory,
IV: dental technicians (*n* = 6).

### 2.4. Polishing the Specimens

All practitioner groups were first introduced to the manufacturer’s recommendations based on a 20-min presentation in which the polishing and the manufacturer’s recommended method were explained. The practitioner groups also received a corresponding handout that they could use during the polishing process. 

Forty-eight monolithic zirconia crowns (two per person) were polished with a two-step zirconia polishing system (Komet Dental, Lemgo, Germany) in two different trials. In step one, a blue zirconia polisher was used (6000 rpm; 94018C 104 055; Komet Dental, Lemgo, Germany). In polishing step two, a white zirconia polisher was used (6000 rpm; 94018F 104 055; Komet Dental, Lemgo, Germany).

During the first trial, the practitioners were not restricted by any time limitation (trial one). The second polishing trial (trial two) was restricted to 15 seconds for each polishing step. The use of magnifying glasses was not required. Every practitioner received a new polishing kit. 

### 2.5. Statistical Analysis

The data were analyzed using the statistics software SPSS (IBM, Armonk, NJ, USA). The data distribution was tested for normality using the Kolmogorov–Smirnov test. Since the data showed non-normal distribution, the non-parametric Mann–Whitney U-Test for the comparison of independent variables was used. The level of significance was set at *p* < 0.05. Differences between the four practitioner groups and differences between the two different trials within each group were evaluated and analyzed with the Wilcoxon signed-rank test. The dataset was illustrated using boxplots. The correlation between the polishing results and the polishing duration was analyzed using the Spearman’s rho test.

## 3. Results

**Figure 1:**
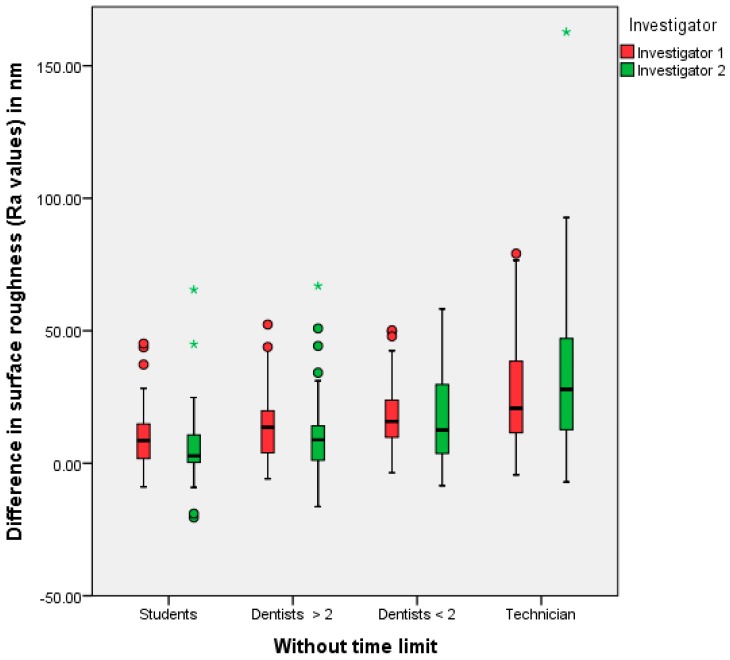
The polishing results (=differences between the Ra values before and after polishing) of the different practitioner groups, investigators one and two/first trial (* extreme values, • outliers).

**Figure 2:**
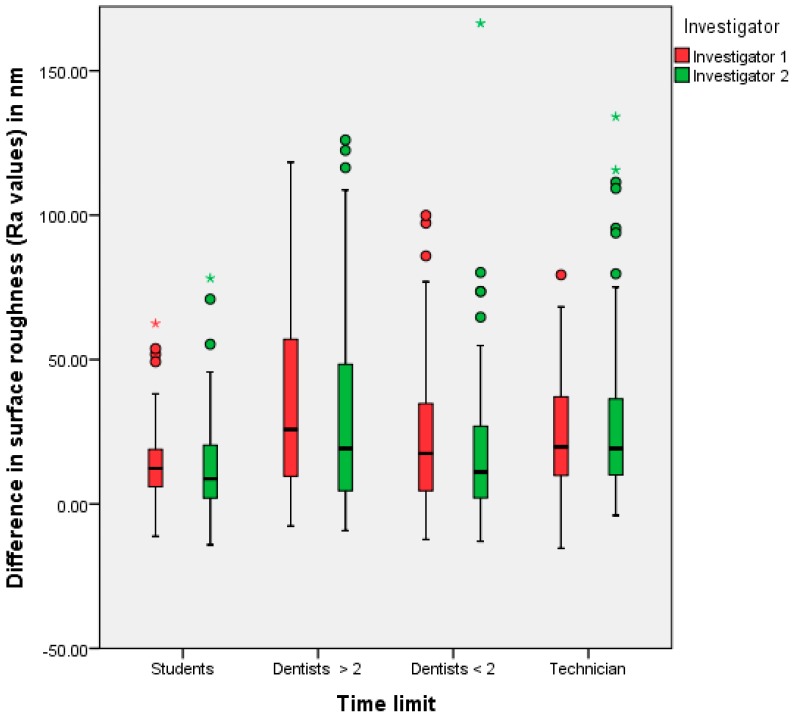
Polishing results (=differences between the Ra values before and after polishing) of the different practitioner groups, investigators one and two/second trial (* extreme values, • outliers).

No statistically significant difference in surface roughness was found between the polishing results of the selected practitioner groups. The statistical results for the first trial (no time limitation) for investigators one and two are summarized in Table 1 and Table 2. A graphical representation of Table 1 and Table 2 is shown in Figure 1. In the first trial, a major difference in surface finish was achieved by the dental technicians. The lowest difference was achieved by the dental students. The second trial showed the greatest median values from experienced dentists, followed by the technicians (see Table 3 and Table 4, and Figure 2).

In the analysis of the two different trials, no significant correlation was found between the polishing time and the results. In summary, the surface roughness was smoothed in each trial after using both polishers, regardless of the practitioner.

## 4. Discussion

The final surface smoothing of dental restorations is an essential procedure after occlusal adjustment [7,9,14,15,16]. Rather than re-glazing or re-polishing in the laboratory, this step can be accomplished chairside in everyday practice. Many different polishing kits are available and have been investigated in a few studies already [14,16,18,23]. Especially for zirconia, different two- or three-step polishing kits have been tested with equal polishing results [18]. Furthermore, von Köckritz et al. investigated the difference between manual and mechanical (by a polishing robot) polishing. Those results showed nearly the same surface effect of manual polishing compared to mechanical polishing [23].

Because little instruction is available from the manufacturer regarding the polishing process in terms of duration, contact pressure, or movement, equilibration between clinicians could not occur. Since no significant correlation was found between the polishing time and the results, the polishing duration may be a negligible parameter. Moreover, in everyday practice, as opposed to clinical investigations, exact adherence to a certain contact pressure cannot easily be measured or controlled.

Regarding the results of this investigation, the level of experience of a practitioner seems to have an influence on the polishing result. However, according to the Mann–Whitney U Test, no significant differences in surface roughness were found between the study participants at different experience levels or for the polishing duration. The dental technicians showed the greatest differences in surface roughness before and after polishing in the first trial. In the second trial, the greatest polishing success was observed in dentists with more than two years of experience. The lowest median values were found in both trials in the group of dental students. This tendency allows for the presumption that dental students are still less experienced in the use of polishing agents and in the handling of ceramics than experienced dental technicians. Therefore, the surface roughness of ceramic crowns after chairside polishing seems to depend on practitioner experience. Regarding the measurements of both investigators, the same trends and similar values were identified. 

The Ra value as a roughness parameter has been used in this investigation, because this is the most commonly used value for roughness determination and it therefore allows an easier comparability to other studies. Consequently, a comparison of individual values would not be conclusive. A smooth crown surface is recognized as a precondition to prevent increased antagonist wear [17,24]. High-gloss polished zirconium has an acceptable physiologic abrasive effect (on steatite as an enamel analogue) [25]. Some investigations analyzed the abrasion behavior of different ceramic materials and examined volume losses in cubic millimeters [15,26,27]. Nonetheless, the exact wear mechanism remains unclear [28,29,30,31,32]. Therefore, focusing scientific attention on the sufficient performance of the available polishing kits and intraoral polishing procedures is important. Although many different polishing kits have been investigated in the past, no explicit declaration has been made concerning a required surface roughness. 

Our study shows no significant differences in the polishing results of different dental professionals. However, a conclusion cannot be drawn on the effect on antagonist wear. A follow-up study could detect the possible abrasion behavior of the zirconia crowns polished by different professionals with the same polishing set. Then, a clinically sufficient polish could be evaluated. 

The clinical relevance of antagonist abrasion in the existing, polished specimens should be proven in further investigations. 

## 5. Conclusions

According to our findings the current polishing set ensures a reliable chairside polish of monolithic zirconium dioxide crowns. If the application protocol is followed, every dental practitioner can achieve a sufficient chairside polish of zirconia crowns. The success of the surface polish is independent of the polishing time. 

This research did not receive any specific grant from funding agencies in the public, commercial, or not-for-profit sectors.

## Figures and Tables

**Table 1:** Descriptive analysis of the Ra values (nm) of investigator 1 and trial one achieved by the specific practitioner groups.

Statistical Characteristics	Ra (nm)			
	Students	Dentists > 2	Dentists < 2	Technicians
25th Percentile	4.70	10.63	10.82	10.15
50th Percentile	6.72	12.23	15.17	25.47
75th Percentile	17.9	16.95	25.75	35.26
Minimum	4.12	9.90	9.97	9.98
Maximum	21.01	29.49	34.52	49.27
Median	6.72	12.23	15.17	25.47

**Table 2:** Descriptive analysis of the Ra values (nm) of investigator 2 and trial one achieved by the specific practitioner groups.

Statistical Characteristics	Ra (nm)			
	Students	Dentists > 2	Dentists < 2	Technicians
25th Percentile	1.48	2.77	6.19	17.75
50th Percentile	5.78	6.35	17.06	30.87
75th Percentile	10.52	16.84	23.98	45.73
Minimum	1.47	2.03	5.70	6.88
Maximum	11.79	22.35	28.58	59.84
Median	5.78	6.35	17.06	30.87

**Table 3:** Descriptive analysis of the Ra values (nm) of investigator 1 and trial two achieved by the specific practitioner groups

Statistical Characteristics	Ra (nm)			
	Students	Dentists > 2	Dentists < 2	Technicians
25th Percentile	9.52	7.77	11.17	13.14
50th Percentile	11.5	41.35	14.47	19.65
75th Percentile	25.27	54.11	32.89	38.73
Minimum	5.62	6.96	6.34	13.02
Maximum	29.6	59.02	63.1	52.86
Median	11.5	41.35	14.47	19.65

**Table 4:** Descriptive analysis of the Ra values (nm) of investigator 2 and trial two achieved by the specific practitioner groups

Statistical Characteristics	Ra (nm)			
	Students	Dentists > 2	Dentists < 2	Technicians
25th Percentile	3.69	9.49	5.20	14.24
50th Percentile	8.28	28.13	16.02	18.96
75th Percentile	22.38	58.78	29.15	47.40
Minimum	3.17	5.90	1.44	12.18
Maximum	36.66	78.76	52.25	89.53
Median	8.28	28.13	16.02	18.69
